# Atypical Mycobacterial Infection Presenting as Persistent Skin Lesion in a Patient with Ulcerative Colitis

**DOI:** 10.1155/2011/480987

**Published:** 2011-10-05

**Authors:** Giorgos Bamias, George L. Daikos, Spyros I. Siakavellas, Garyfallia Kaltsa, Stavroula Smilakou, Ioannis Katsogridakis, Irene Vafiadis-Zouboulis, Spiros D. Ladas

**Affiliations:** ^1^Gastroenterology Division, 1st Department of Internal Medicine-Propaedeutic, Laikon General Hospital, University of Athens, 17 Agiou Thoma Street, 11527 Athens, Greece; ^2^Department of Clinical Microbiology, Laikon General Hospital, 17 Agiou Thoma Street, 11527 Athens, Greece

## Abstract

Immunosuppressive drugs are commonly used for the treatment of inflammatory bowel disease. Patients receiving immunosuppressants are susceptible to a variety of infections with opportunistic pathogens. We present a case of skin infection with *Mycobacterium chelonae* in a 60-year-old Caucasian woman with ulcerative colitis who had been treated with corticosteroids and azathioprine. The disease manifested with fever and rash involving the right leg. Infliximab was administered due to a presumptive diagnosis of pyoderma gangrenosum, leading to worsening of the clinical syndrome and admission to our hospital. Routine cultures from various sites were all negative. However, Ziehl-Neelsen staining of pus from the lesions revealed acid-fast bacilli, and culture yielded a rapidly growing mycobacterium further identified as *M. chelonae*. The patient responded to a clarithromycin-based regimen. Clinicians should be aware of skin lesions caused by atypical mycobacteria in immunocompromised patients with inflammatory bowel disease. Furthermore, they should be able to thoroughly investigate and promptly treat these conditions.

## 1. Introduction

Immunosuppressive drugs, consisting of corticosteroids, immunomodulators, and biological factors, are the mainstay of treatment for inflammatory bowel disease (IBD). These medications lead to a state of immunosuppression, the severity of which depends upon the specific drug, the dose administered, and the duration of treatment [1]. Patients receiving immunosuppressants become susceptible to infections, caused not only by common pathogens, but also, by various opportunistic microorganisms. The latter may cause unusual clinical syndromes, thus generating diagnostic and therapeutic challenges, as they are easily confused with the variety of extraintestinal manifestations of Ulcerative Colitis (UC) and Crohn's disease (CD) [2]. Herein, we describe a case of skin infection with *Mycobacterium chelonae* in a woman who was under long-term immunosuppressive treatment for UC. The lesion was initially misdiagnosed as pyoderma gangrenosum resulting in initiation of anti-TNF treatment. The final diagnosis was achieved only when microbiological testing with optimal culture conditions was applied.

## 2. Case Presentation

A 60-year-old female, with a 12-year history of UC involving the entire colon, was admitted to our hospital for evaluation of skin lesions and low-grade fever. The patient had been receiving 5-ASA preparations, since UC diagnosis, and corticosteroids and azathioprine for the last 3 years. At admission, UC was in clinical remission and the patient was on 16 mg of methylprednisolone and 150 mg azathioprine daily. 

Four months prior to admission, while intestinal disease was in remission, the patient noticed several painless, erythematous nodules on her right lower extremity. There was no history of prior trauma. Local corticosteroids had no beneficial effect. A month and a half later, low-grade fever (up to 37.6°C) was noticed. An incision was made in one of the lesions, pus obtained and sent for culture, which grew *Staphylococcus aureus*. Two-week treatment with doxycycline resulted in no clinical improvement. The diagnosis of pyoderma gangrenosum (with possible bacterial contamination) was considered and treatment with infliximab initiated in a classical induction scheme (0–2–6 wks). Before starting therapy with infliximab, a tuberculin skin test was performed and reported negative, whereas a chest X-ray was unremarkable. Ten days after the second infusion of infliximab, the patient noticed worsening of the skin lesions along with high fever (38.7°C) and was admitted to our hospital.

**Figure 1:**
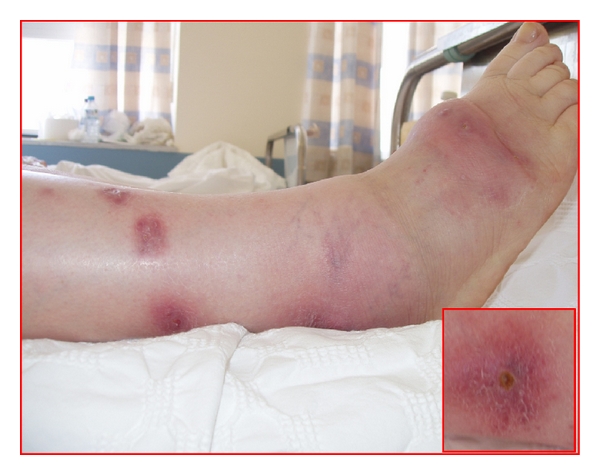
Multiple violet skin lesions in the right lower extremity of the patient. Pitting edema of the ankle and foot is also demonstrated. The inlet depicts one of the lesions, where an orifice leading to a pus collection is shown.

Clinical examination revealed an obese patient (BMI: 32) with temperature of 37.7°C. Inspection of the lower extremities showed multiple, painless, erythroviolet, round, and clearly delineated nodular lesions, 2–5 cm in size, which were localized in the lower right leg and foot (Figure 1). There was seropurulent discharge from some of the lesions. Laboratory investigations revealed mild leukocytosis (WBC: 11,900 k/*μ*L, P: 85%, L: 7%), with elevated erythrocyte sedimentation rate (60 mm/hr, normal range 0–20 mm/hr) and C-reactive protein (12.6 mg/dL, normal range 0–5 mg/dL) with no abnormalities on biochemical and urine tests. 

Colonoscopy revealed endoscopic features consistent with quiescent ulcerative colitis, which was confirmed by histological examination of colon biopsies that showed absence of active inflammation and signs of chronic injury with disturbed mucosal architecture.

As the patient experienced persistent fever (37.8 to 38.2°C), we performed extensive search for infectious agents. Blood, urine, and stool cultures as well as chest X-rays were repeatedly negative. Tuberculin skin test was negative and CT-scan of the lower abdomen was unremarkable. MRI scan of the lower right limb and three-phase bone scintigraphy with ^99m^Tc-MDP revealed signs consistent with inflammation of the skin and subcutaneous tissue but no evidence of bone involvement or abscess formation. A skin biopsy showed findings consistent with a granulomatous inflammatory process of the skin, without necrosis.

**Figure 2:**
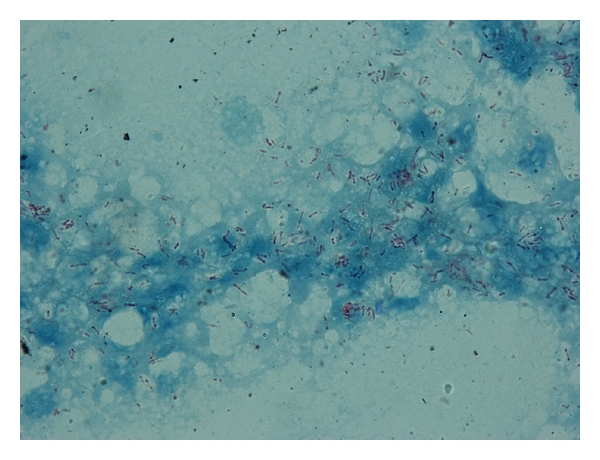
Ziehl-Neelsen staining of pus obtained from a draining lesion reveals multiple acid-fast bacilli.

**Figure 3:**
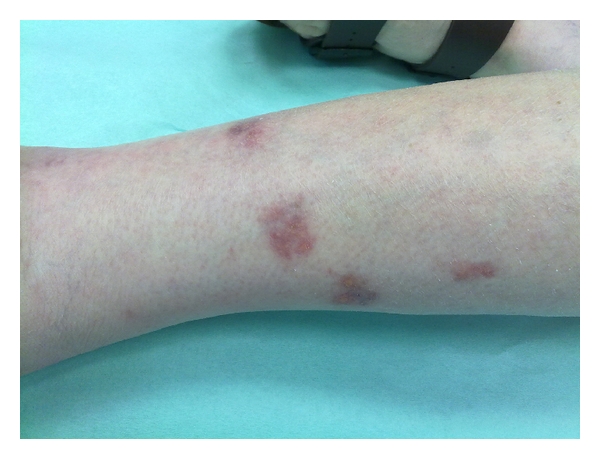
Photograph taken 6 months after treatment shows healing of the skin lesions with complete cessation of discharge and residual discoloration.

Pus was collected from one of the draining lesions. Direct microscopic examination after Ziehl-Neelsen staining was positive for acid-fast bacilli (Figure 2). Cultures of the purulent material on both blood agar and Lowenstein media yielded a rapidly growing, nontuberculous mycobacterium. Treatment was initiated with intravenous cefoxitin 3 g qid, clarithromycin 500 mg bid, and amikacin 1 g qd. Two weeks later the patient was significantly improved with cessation of fever and diminishing of cutaneous lesions. Following the identification of the microorganism as *Mycobacterium chelonae *in a reference laboratory and susceptibility testing, she was discharged home on clarithromycin 500 mg bid for an additional six months. Administration of infliximab and azathioprine was terminated and corticosteroids rapidly tapered and discontinued. Upon several follow-up visits, the patient remained afebrile and the cutaneous lesions were gradually eliminated (Figure 3).

## 3. Discussion

The present case illustrates the difficulties in managing dermatological manifestations in patients with IBD. The differential diagnosis should include not only those skin conditions that occur more frequently in IBD (most notably erythema nodosum and pyoderma gangrenosum) but also treatment-associated lesions [3]. Among the latter, skin infections should always be considered in patients who receive immunosuppressive regimens, particularly in the setting of a new rash and fever [2]. Such infections accounted for 21% of all and 40.5% of serious infections in a large series of patients receiving anti-TNF agents [4]. 

In recent years, the risk for immunosuppression-induced reactivation of tuberculosis has been well documented and led to pretreatment testing for latent infection and prophylactic chemotherapy of individuals at risk [5]. On the other hand, infections with atypical mycobacteria are less recognized outside the infectious diseases specialty [6]. Nevertheless, studies from large databases of patients on immunosuppressants have reported one case of atypical mycobacterial infection for every 4–10 cases of tuberculosis [7]. Interestingly, a recent survey reported twice as many infections with nontuberculous mycobacteria as compared to cases of tuberculosis in patients receiving anti-TNF agents for various conditions [8]. Similar to our patient, nontuberculous mycobacterial disease frequently localizes to the skin and soft tissues [9]. The microorganism responsible for the skin infection in our patient was *M. chelonae*. To our knowledge, no published report of such infection exists in the IBD literature. However, in recent studies in anti-TNF-treated patients with various conditions, *M. chelonae* accounted for 10% of all mycobacterial or 4% of non-tuberculous mycobacterial infections, respectively [8, 9]. In the IBD population, infections with atypical mycobacteria such as *M. gordonae*, *M. fortuitum*,* M. marinum*, and *M. xenopi* have been reported [10–12].

Recently, Toruner et al. studied the presentation of and risk factors for opportunistic infections among IBD patients [10]. Although all immunosuppressive drugs (corticosteroids, immunomodulators, biologicals) increased the risk of infection, this risk was disproportionally elevated when combinations of drugs were administered. Caution, therefore, is needed when prescribing such medications and regular reassessment is required in order to discontinue any unnecessary therapies. This is of particular relevance to the present case, as our patient was treated with corticosteroids for an extended period, before admission to our unit. This, in combination to azathioprine administration, most probably led to the development of mycobacterial infection. The ability to discontinue both medications after the diagnosis of *M. chelonae* infection, without a flare in disease activity, emphasizes the need for constant cost/benefit evaluation in the treatment of IBD patients. In particular, current guidelines for the treatment of IBD clearly state that prolonged administration of corticosteroids to maintain remission is not an acceptable option. Corticosteroid-sparing agents such as thiopurines and methotrexate should be used followed by early introduction of biologicals in treatment failures. Furthermore, the development of a febrile skin lesion in our patient was mistakenly considered as pyoderma gangrenosum, a diagnosis which led to infliximab administration and the eventual unmasking of the mycobacterial infection. Nevertheless, the occurrence of pyoderma gangrenosum in our patient was highly unlikely, given the corticosteroid use and the quiescent phase of colitis. Had a colonoscopy been performed in our patient before the initiation of infliximab, it might have helped therapeutic decisions. If no active inflammation were seen, it might have questioned the diagnosis of pyoderma gangrenosum and prompted early consultation by a dermatologist and/or infectious diseases specialist. In fact, such an approach is strongly recommended by current guidelines on the management of infectious complications in IBD patients receiving immunosuppressants [2]. This is of particular importance as skin infections, including those caused by atypical mycobacteria, can easily be confused with the various dermatological manifestations of IBD. Similar to the present case, the initial clinical impression for these lesions has been reported very often to be misleading [13]. In addition, gastroenterologists may not be familiar with the specific conditions required for the growth of these microorganisms in culture. In our case, initial cultures were performed in regular media and reported negative, as no growth was observed after incubation for 48 hours. Nevertheless, when the possibility of atypical mycobacteria was considered, cultures were left to incubate for several days, resulting to the growth of *M. chelonae*. Indeed, it has been recently suggested that infection with non-tuberculous mycobacteria must be suspected when routine microbiological tests are negative [14].

## 4. Conclusion

We present a rare case of skin infection with *M. chelonae* in a patient with IBD. It is important that clinical gastroenterologists recognize atypical mycobacterial infections in this group of patients and be able to thoroughly investigate and aggressively treat those conditions.
